# A first look at childhood abuse in women with obstructive sleep apnea

**DOI:** 10.3389/frsle.2023.1281425

**Published:** 2024-01-08

**Authors:** Amrita Pal, Fernando Martinez, Jennifer Wagman, Ravi S. Aysola, Ari Shechter, Vincent Mysliwiec, Jennifer L. Martin, Paul M. Macey

**Affiliations:** ^1^UCLA School of Nursing, University of California, Los Angeles, Los Angeles, CA, United States; ^2^UCLA Fielding School of Public Health, University of California, Los Angeles, Los Angeles, CA, United States; ^3^Department of Medicine, David Geffen School of Medicine at UCLA, University of California, Los Angeles, Los Angeles, CA, United States; ^4^Division of Cardiology, Columbia University Irving Medical Center, New York, NY, United States; ^5^Department of Sleep Medicine, University of Texas Health Science Center San Antonio, San Antonio, TX, United States; ^6^Department of Medicine, VA Greater Los Angeles Health Care, Los Angeles, CA, United States

**Keywords:** sleep-disordered breathing, obesity, trauma, sexual abuse, postmenopausal women

## Abstract

**Study objectives:**

Women who experienced childhood sexual abuse have higher rates of obesity, a risk factor for obstructive sleep apnea (OSA). We assessed if prior childhood sexual abuse was more common in women with OSA vs. those in the control group, with possible mediation by obesity.

**Methods:**

In a secondary analysis of a larger project, we studied 21 women with OSA (age mean ± *SD* 59 ± 12 years, body mass index [BMI] 33 ± 8 kg/m^2^, respiratory event index [REI] 25 ± 16 events/hour, and Epworth Sleepiness Scale [ESS] score 8 ± 5) and 21 women without OSA (age 53 ± 9 years, BMI 25 ± 5 kg/m^2^, REI [in 7/21 women] 1 ± 1 events/hour, and ESS score, 5 ± 3). We evaluated four categories of trauma (general, physical, emotional, and sexual abuse) with the Early Trauma Inventory Self-Report–Short Form (ETISR-SF). We assessed group differences in trauma scores with independent samples *t*-tests and multiple regressions. Parametric Sobel tests were used to model BMI as a mediator for individual trauma scores predicting OSA in women.

**Results:**

Early childhood sexual abuse reported on the ETISR-SF was 2.4 times more common in women with vs. without OSA (*p* = 0.02 for group difference). Other trauma scores were not significantly different between women with and without OSA. However, BMI was a significant mediator (*p* = 0.02) in predicting OSA in women who experienced childhood physical abuse.

**Conclusion:**

Childhood sexual abuse was more common in women with vs. without OSA. BMI was a mediator for OSA of childhood physical but not sexual abuse. This preliminary hypothesis-generating study suggests that there may be physiological impacts of childhood trauma in women that predispose them to OSA.

## Introduction

Although less common in women than men, obstructive sleep apnea (OSA) affects an estimated 17% of adult females (Tietjens et al., 2019), and there may be sex-specific factors contributing to this occurrence. In particular, childhood sexual abuse is experienced more frequently by women than men and can increase the risk of developing many sleep disorders (Krakow et al., 2000, 2002; Greenfield et al., 2011; Rich-Edwards et al., 2012; Steine et al., 2012; Jaoude et al., 2014; Lind et al., 2016; McWhorter et al., 2019). Sexual abuse is the most prevalent childhood abuse in women, with a reported prevalence of over 20% in the United States (Moody et al., 2018). There is evidence of a link between sexual abuse and OSA (Krakow et al., 2002, 2015; Rich-Edwards et al., 2012), raising the question of whether women with OSA are more likely to have experienced childhood sexual abuse. Given that obesity is also another well-established risk factor for OSA (Jehan et al., 2017), a possible underlying mechanism could be childhood trauma-associated weight gain (Gooding et al., 2015), which is also common in women (Felitti, 1993; Noll et al., 2007; Midei et al., 2010; Smith et al., 2010; Richardson et al., 2014; Gooding et al., 2015). The link between childhood sexual abuse and obesity is well established, as is the obesity–OSA connection; the question is whether childhood sexual trauma is an independent risk factor for OSA, specifically in women.

The mechanisms by which obesity leads to OSA are well studied (Tuomilehto et al., 2013). Obesity can lead to fat deposition in the upper airway, potentially impairing muscle tone. Obesity-related metabolic activity of adipose tissue may promote inflammatory cytokines that may increase pharyngeal collapsibility through mechanical effects on pharyngeal soft tissues. There are also likely effects on lung volume and altered signaling of airway neuromuscular nerves (Schwartz et al., 2008; Sousa et al., 2008), with, for example, reduced muscle tone possibly due to damage to the genioglossus nerve from mitochondrial dysfunction elicited by an obesity-related diet (Karimi et al., 2010; Chen et al., 2021). These pathophysiologies can lead to more upper airway collapsibility, which is a proximal cause of obstructive apneas (Gold, 2011).

The objective was to examine the association between childhood abuse and adult onset of OSA based on a secondary analysis of data from an ongoing study. We focused on women given the sex-specific characteristics of sexual abuse trauma and OSA. We theorized that female sexual abuse survivors have a higher rate of OSA than females without a history of sexual abuse. We could not address this theory directly because the existing study did not assess women with and without a history of sexual abuse. However, if the theory is true, we would predict that women with OSA would report a higher incidence of childhood sexual abuse than comparable women without OSA, so we tested that hypothesis in this secondary analysis. We also hypothesized that body mass index (BMI) would mediate any relationship between trauma and OSA.

## Methods

For this secondary analysis, we selected 21 women with OSA and 21 control women participants from a larger study conducted between 2013 and 2022 on sex-specific effects of OSA's impact on the brain. Recruitment was in the Los Angeles area via physical and online flyers, and we targeted adults with and without diagnosed or suspected OSA. Participants were screened to exclude non-OSA diagnosed sleep and current mental health disorders, major illness or traumatic brain injury, stroke, major cardiovascular disease, and recent (<3 months) use of psychotropic medications. Participants with OSA were diagnosed using a two-night home sleep apnea test (HSAT) or an in-lab overnight polysomnography (PSG) at the University of California, Los Angeles (UCLA) Sleep Disorders Center. HSAT testing was conducted with an ARES™ device (Ayappa et al., 2008), which includes electroencephalogram measurements for sleep staging, with scoring based on the 2012 American Academy of Sleep Medicine criteria (Berry et al., 2019). For a diagnosis of OSA, participants either had a respiratory event index [REI] of ≥5 events/hour on the HSAT or an apnea–hypopnea index of ≥5 events/hour on the PSG. Among the OSA participants, 42% used continuous positive airway pressure (CPAP). Female control participants were selected to match the age range of the OSA group and ensure a non-significant difference in mean ages by independent samples *t*-test (threshold *p* = 0.05). For the control group, participants who screened positive for potential OSA based on the reported symptoms of OSA (excessive daytime sleepiness, snoring, and nighttime awakening out of breath) were referred for an HSAT to confirm the absence of OSA. Borderline cases of OSA in the control group were excluded [respiratory disturbance index (RDI)/apnea hypopnea index (AHI) 4–6 events/hour]. All participants visited UCLA, where their height and weight were measured for BMI calculation and their menopausal status was recorded (categorized as pre, peri, or post). They completed anxiety, self-reported menopausal status (pre, peri, or post), and trauma questionnaires in a private setting using encrypted online survey tools. All procedures were approved and performed according to the relevant guidelines and regulations of the UCLA institutional review board. All participants provided written informed consent. All data generated or analyzed during this study are available in the associated online data repository (Macey, 2023).

Trauma was measured with the Early Trauma Inventory Self-Report–Short Form (ETISR-SF) (Bremner et al., 2007; Hörberg et al., 2019). The ETISR-SF measures four categories of trauma on continuous scales: general trauma, physical abuse, emotional abuse, and sexual abuse (Bremner et al., 2007). Based on reported normative scores (Bremner et al., 2007), we categorized trauma occurrence as higher than the threshold values of 1.6 for general trauma, 1.0 for physical abuse, 0.8 for emotional abuse, and 0.2 for sexual abuse (Bremner et al., 2007). Anxiety was measured with the Generalized Anxiety Disorder Scale-7 Item (GAD-7), a short self-report measure (Spitzer et al., 2006). The GAD-7 scale ranges from 0 to 21, and scores of 5, 10, and 15 are traditionally taken as the cutoff points for mild, moderate, and severe anxiety, respectively. When used as a screening tool, further evaluation is recommended when the score is 8 or greater (Kroenke et al., 2007).

Independent samples *t*-tests were run to compare trauma scores, BMI, and anxiety between the OSA and control groups. We conducted a multiple regression analysis using the four subcategories of trauma scores as predictors of OSA status. We modeled BMI as mediating each of the four trauma scores on OSA diagnosis in a non-parametric bootstrap mediation test by Efron (Efron and Tibshirani, 1994; Fritz and Mackinnon, 2007) to determine the indirect effect of childhood trauma scores on OSA with BMI as a mediator. This test was conducted with SPSS macro PROCESS v3.5, software written by Andrew F. Hayes (Preacher and Hayes, 2008). The number of bootstrap samples for percentile bootstrap confidence intervals was 5,000.

We report the confidence interval or *p*-value as appropriate, and we set the significance threshold as within 95% confidence intervals or *p* ≤ 0.05.

## Results

The women in the OSA group were slightly older than those in the control group (*p* = 0.08) and had significantly higher BMIs (Table 1). The four women in the control group who were screened for OSA had a mean REI of 1 event/hour. The other 17 women in the control group who did not have an HSAT were screened as having a low probability of OSA. The excessive daytime sleepiness ESS score for OSA was moderately higher than for control group (8 vs. 5, respectively, *p* = 0.02). Anxiety was higher in the OSA group, but the means of both groups were below the cutoff for mild anxiety symptoms (mean GAD-7: OSA 4 vs. control 1, *p* = 0.01). The menopausal status was distributed similarly across both groups (*p* = 0.8).

**Table 1:** Participant characteristics.

**A: Clinical measures mean ±*SEM***	**Age years**	**BMI kg/m^2^**	**Anxiety GAD-7**	**REI events/hour**	**Oxygen saturation nadir %**	**Excessive daytime sleepiness ESS**	**Menopause**
Control (*N* = 21)	53.1 ± 9.1	25.1 ± 5.0	1.0 ± 1.2	1.0 ± 0.8 (*N* = 4)	89.0 ± 2.5 (*N* = 4)	4.7 ± 3.5	2 pre, 5 peri, 14 post
OSA (*N* = 21)	59.0 ± 12.9	33.0 ± 7.6	3.7 ± 3.9	24.7 ± 15.8	81.5 ± 9.1	8.0 ± 4.8	1 pre, 4 peri, 16 post
*p*-value (*t*-test or chi-square)	*0.08*	* <0.001*	*0.006*	* <0.001*	*0.004*	*0.02*	0.8
**B: ETISR-SF scores mean** ±***SEM***	**General**	**Physical**	**Emotional**	**Sexual**
Control (*N* = 21)	2.62 ± 2.13	0.90 ± 1.34	1.57 ± 1.83	0.48 ± 0.98
OSA (*N* = 21)	2.62 ± 2.38	1.57 ± 1.47	1.81 ± 1.94	1.62 ± 1.91
*p*-value (*t*-test)	0.99	0.1	0.7	*0.02*

A: Clinical characteristics. The REI and nadir and mean peripheral capillary oxygen saturation (SpO_2_) scores in control were only available in 4 of 21 women. B: Percentage of women in each group with trauma, and the mean ± standard error of mean of the trauma scores and clinical variables. *p*-values reflect group differences based on the chi-square text for menopause and independent samples *t*-tests for other variables (italicized for *p* ≤ 0.05). SEM, standard error of mean; BMI, body mass index; GAD-7, Generalized Disorder Scale−7 Item; REI, respiratory event index; ESS, Epworth Sleepiness Scale; OSA, obstructed sleep apnea; ETISR-SF, Early Trauma Inventory Self-Report–Short Form.

**Figure 1:**
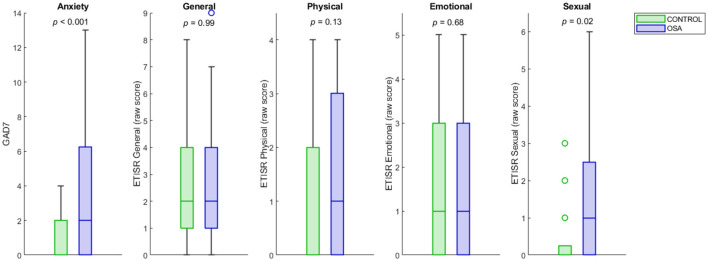
OSA and control distributions of anxiety and childhood trauma scores. The OSA and control distributions of GAD-7 and childhood sexual, physical, emotional and general abuse trauma scores are shown as box plots. The *y*-axes represent raw scores on the GAD-7 and ETISR-SF trauma subscales. The center line of each bar is the median, and the upper and lower bounds represent the upper and lower quartiles. Outliers are shown as circles. The group difference *p*-values for independent samples *t*-tests are under each title. OSA, obstructive sleep apnea; GAD-7, Generalized Disorder Scale−7 Item; ETISR-SF, Early Trauma Inventory Self-Report–Short Form.

Sexual abuse scores were significantly higher in women with OSA compared to women without OSA 0.48 ± 0.98 (1.6 vs. 0.5, respectively, *p* = 0.02; Table 1; Figure 1). Other childhood trauma categories did not show significant differences. Sexual abuse was a significant predictor of an OSA diagnosis (unstandardized regression coefficient = 0.11, standard error = 0.048, *p* = 0.03; Table 2). Other abuse scores did not show significant group differences in means or as predictors. Using GPower *post-hoc* analysis (Faul et al., 2007), the effect size for the multiple regression model was *f*^2^ = 0.51.

**Table 2:** Model of trauma types predicting OSA diagnosis.

**Overall model**	**Sum of squares**	***df***	**Mean square**	***F***	**Sig**.
Regression	2.142	4	0.536	2.371	0.07
Residual	8.358	37	0.226		
Total	10.5	41			
**Model variables**	**Unstandardized coefficients**		**Standardized coefficients**	***T***	**Sig**.
	***B***	**Std. Error**	**Beta**		
Sexual	0.111	0.048	0.352	2.296	*0.027*
General	0.053	0.041	0.232	1.291	0.205
Physical	0.128	0.076	0.362	1.698	0.098
Emotional	0.035	0.054	0.129	0.647	0.522

Trauma scores in the general, physical, emotional, and sexual categories as predictors in a multiple regression model of OSA diagnosis (OSA or control) in women. Significant (*p* ≤ 0.05) relationships are indicated in italics. OSA, obstructive sleep apnea.

**Figure 2:**
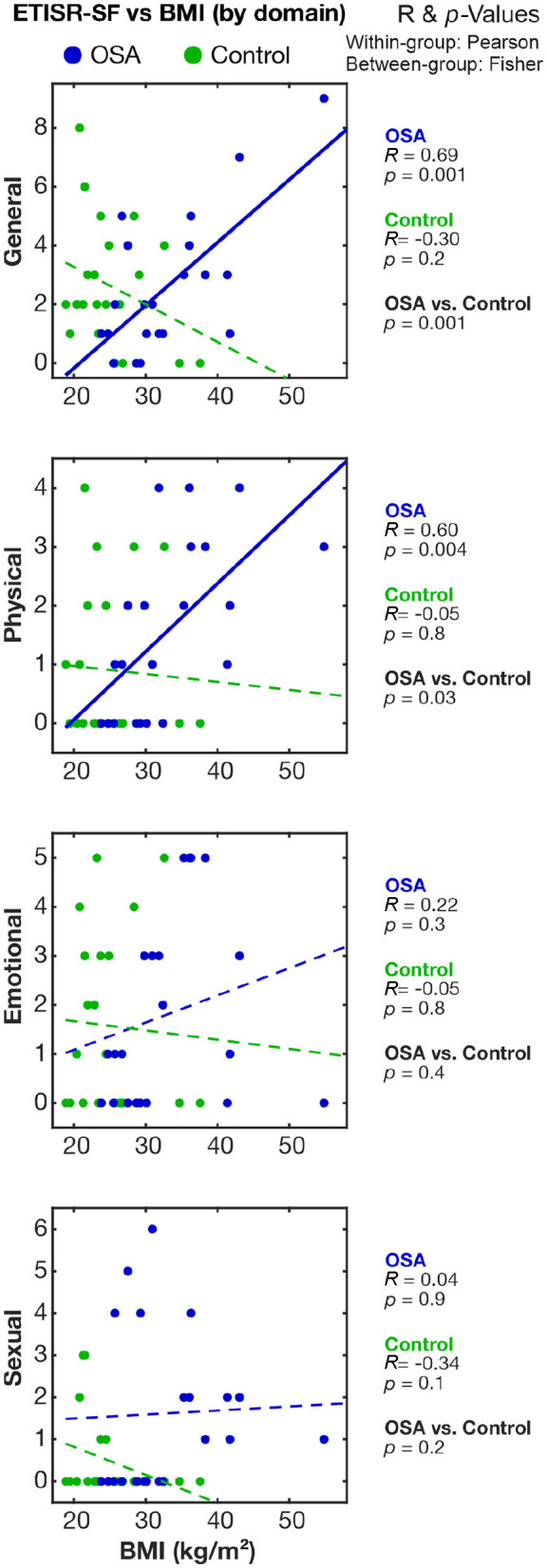
Correlations between childhood sexual trauma and BMI in the OSA and control groups. The relationships between childhood trauma scores and BMI in women with OSA and those in the control group are shown on scatterplots. Correlation and associated *p*-values values are shown on the right; within-group (OSA, Control) are Pearson's *R* values and the significance of correlation; and between-group (OSA vs. Control) significance is based on Fisher's *z* comparison of correlations. BMI, body mass index; OSA, obstructive sleep apnea; ETISR-SF, Early Trauma Inventory Self-Report–Short Form.

The correlation of ETISR-SF domain scores with BMI showed positive Pearson's *R* values in OSA with the general and physical categories but not the emotional or sexual categories (Figure 2). Both general and physical ETISR-SF correlations were significantly different between women with OSA and those in the control group (Figure 2). The full correlation tables and raw data are available at the data repository for this study (Macey, 2023).

We conducted Efron's non-parametric bootstrapping mediation test with the following results. The overall model of OSA with BMI and sexual abuse (SA) as predictors was significant at *p* <0.001, with a McFadden score of 0.29. There was a direct positive interaction of SA on BMI, indicating higher sexual abuse in women was associated with higher BMI. The direct effect of BMI on OSA is positive and significant at *b* = 0.19, *SE* = 0.06, 90% CI [0.10, 0.30], *p* = 0.001, indicating, as expected, that higher BMI is predictive of OSA. The direct effect *c'* of SA on OSA was 0.39, 90% CI [0.01, 0.77], *p* = 0.09, *SE* = 0.23; and the indirect effect *c* was 0.25, 90% CI [0.07, 0.61], *SE* = 0.18. Since zero fell outside the confidence interval, the indirect effect was inferred to be non-zero and significant. BMI was a significant (*p* = 0.02) mediator only of childhood physical abuse on OSA diagnosis. BMI was not a significant mediator of emotional abuse or general trauma for an OSA diagnosis. In contrast with our hypothesis, BMI was not a significant mediator of childhood sexual abuse for an OSA diagnosis.

## Discussion

We found that a sample of women with OSA had a higher rate of childhood sexual abuse than women without OSA. While sexual abuse is not typically associated with sleep-disordered breathing, our study suggests that trauma exposure in childhood may be a risk factor for the occurrence of OSA. Additionally, we found that BMI was a mediator of childhood physical abuse (but not sexual or other abuse) on the occurrence of OSA in older women. Thus, in contrast to our hypothesis, BMI did not mediate the association between childhood sexual abuse and OSA, although such relationships may be identifiable in larger samples and with more detailed assessments of childhood experiences.

Factors other than obesity that could link OSA and sexual abuse are neuroendocrine effects of aging in women, including reduced progesterone levels (Stavaras et al., 2012), effects that are exacerbated in sexual abuse survivors who report symptoms of trauma (Smith et al., 2010). Brain circuitry changes may also lead to changes in breathing and cardiovascular control (George Zaki Ghali, 2020), particularly in women (Rich-Edwards et al., 2012; Zoldbrod, 2015). Poor autonomic regulation, including decreased heart rate variability, has been found in both women with early childhood sexual abuse history (Lorenz et al., 2015) and OSA (Park et al., 2016; Pal et al., 2021a); this autonomic dysfunction may extend to breathing control.

Studies by Krakow et al. (2001a, 2002, 2013) show an association with trauma and principally mild OSA, typically comorbid with insomnia (Ong and Crawford, 2013). Comorbid insomnia and OSA, noted by Krakow et al. in their cohort of female sexual abuse survivors (Krakow et al., 2002, 2013), have only recently received attention in terms of a joint diagnosis, for example, as reported by Brock and Mysliwiec (2018) and Sweetman and Lack (2019). Women who are sexual abuse or other trauma survivors may be considered more likely to have insomnia, but sleep problems may be less likely to be attributed to OSA, potentially contributing to an underdiagnosis of sleep-disordered breathing in these women (Lastra and Attarian, 2018). OSA is associated with increased BMI (Tuomilehto et al., 2013), increased anxiety (Wong et al., 2021), and increased overall sympathetic activation (Pal et al., 2021b), especially in women. From our study, we think that the increased sympathetic activation in childhood could have contributed to the occurrence of OSA later in life as the symptoms are similar in both childhood abuse and OSA.

These findings should be considered preliminary and interpreted considering several limitations. This is a cross-sectional study based on secondary data analysis. The exclusion criteria of no current use of psychotropic medications meant that women with severe trauma-related anxiety who need ongoing treatment would have been excluded, thus potentially leading to a lower effect in this study than in the general female OSA population. The sample size was small for mediation analysis, so the lack of a significant BMI effect is not a definitive test of the presence or absence of this pathway. Furthermore, BMI may have been influenced by CPAP usage in the 42% of patients using that treatment. While there is one study showing weight loss in 119 patients starting CPAP (Pociene et al., 2019), most literature shows weight gain to be associated with CPAP use (Drager et al., 2015), including specifically in women (Redenius et al., 2008). Therefore, CPAP may have influenced BMI in some patients, with the direction usually being toward weight gain. Presumably this CPAP influence would confound the mediation analysis in a way that lowers the sensitivity to trauma effects. Thus, it would give slightly greater confidence in the finding of significant BMI mediation of physical trauma influences and slightly less confidence in the lack of a significant BMI influence on sexual trauma influences. The control group's sleep status may not be fully accurate because some women in the control group who were low in clinical probability for OSA did not undergo an HSAT, and a negative HSAT does not conclusively rule out OSA. Insomnia and nightmares were not assessed here, and any sleep disorder other than OSA was an exclusion criterion; because several sleep conditions have been associated with sexual abuse, we recommend assessing all sleep disorders in future studies on this topic (Krakow et al., 2000, 2001b).

In conclusion, we found that a sample of women with OSA had a higher rate of childhood sexual abuse trauma compared with those in an age-matched healthy group. This preliminary study raises the possibility that childhood sexual abuse may place women at higher risk for developing OSA (Plechner, 2004), but follow-up in larger samples is required to establish whether and how pathways from childhood sexual abuse trauma to OSA occur.

## Data availability statement

The original contributions presented in the study are included in the article/Supplementary material, further inquiries can be directed to the corresponding author.

## Ethics statement

The studies involving humans were approved by University of California Los Angeles Institutional Review Board. The studies were conducted in accordance with the local legislation and institutional requirements. The participants provided their written informed consent to participate in this study.

## Author contributions

AP: Conceptualization, Data curation, Formal analysis, Funding acquisition, Investigation, Methodology, Software, Visualization, Writing – original draft, Writing – review & editing. FM: Data curation, Project administration, Visualization, Writing – review & editing. JW: Conceptualization, Writing – review & editing. RA: Methodology, Conceptualization, Resources, Validation, Writing – review & editing. AS: Validation, Writing – review & editing. VM: Conceptualization, Validation, Writing – review & editing. JM: Funding acquisition, Validation, Writing – review & editing. PM: Conceptualization, Data curation, Formal analysis, Funding acquisition, Investigation, Methodology, Project administration, Resources, Supervision, Visualization, Writing – review & editing.
